# Decoding the Temporal Dynamics of Covert Spatial Attention Using Multivariate EEG Analysis: Contributions of Raw Amplitude and Alpha Power

**DOI:** 10.3389/fnhum.2020.570419

**Published:** 2020-10-09

**Authors:** Andrea Desantis, Adrien Chan-Hon-Tong, Thérèse Collins, Hinze Hogendoorn, Patrick Cavanagh

**Affiliations:** ^1^Département Traitement de l’Information et Systèmes, ONERA, Palaiseau, France; ^2^Integrative Neuroscience and Cognition Center (UMR 8002), CNRS and Université de Paris, Paris, France; ^3^Institut de Neurosciences de la Timone (UMR 7289), CNRS and Aix-Marseille Université, Marseille, France; ^4^Melbourne School of Psychological Sciences, The University of Melbourne, Melbourne, VIC, Australia; ^5^Department of Experimental Psychology, Helmholtz Institute, Utrecht University, Utrecht, Netherlands; ^6^Department of Psychological and Brain Sciences, Dartmouth College, Hanover, NH, United States; ^7^Department of Psychology, Glendon College, North York, ON, Canada

**Keywords:** EEG decoding, multivariate pattern classification, covert spatial attention, raw activity, alpha oscillations

## Abstract

Attention can be oriented in space covertly without the need of eye movements. We used multivariate pattern classification analyses (MVPA) to investigate whether the time course of the deployment of covert spatial attention leading up to the observer’s perceptual decision can be decoded from both EEG alpha power and raw activity traces. Decoding attention from these signals can help determine whether raw EEG signals and alpha power reflect the same or distinct features of attentional selection. Using a classical cueing task, we showed that the orientation of covert spatial attention can be decoded by both signals. However, raw activity and alpha power may reflect different features of spatial attention, with alpha power more associated with the orientation of covert attention in space and raw activity with the influence of attention on perceptual processes.

## Introduction

The visual world conveys more information than observers can process (James, 1890; Moran and Desimone, 1985) and attention is the mechanism that allow us to select and prioritize visual information based on our goals (Carrasco, 2011). Normally, attention is focused on our central vision and moves in tandem with our eye-movements. However, it can also be oriented in space covertly without moving our eyes (Posner, 1980); we can “look out of the corner of our eye” as popular wisdom would say. Covert spatial attention has been extensively studied and one of the most popular methods used to manipulate it consists of cueing the location where a target stimulus will appear. Observers are faster to report the presentation of cued compared to uncued targets (Posner, 1980). Further studies have also shown that covert attention does not only enhance processing time but also increase contrast sensitivity (Ling and Carrasco, 2006).

While overt attention is bound to eye-movements, researchers suggested that covert attention operates before the execution of saccades (Deubel and Schneider, 1996). It facilitates the processing of information presented in visual periphery at the location where the eyes are about to be directed, and ultimately guides the preparation and execution of saccades (Hoffman and Subramaniam, 1995; de Haan et al., 2008; Zhao et al., 2012; Casteau and Smith, 2020).

There are two types of attention mechanisms that guide covert orienting and the selection of sensory information: exogenous and endogenous mechanisms. Notably, we can decide to voluntarily (i.e., endogenously) orient our attention to a given location in space, or our attention can be automatically and involuntarily (i.e., exogenously) attracted by a salient stimulus in the environment (Posner and Cohen, 1984). These two kinds of covert orienting systems exhibit similar effects on perception but they differ with respect to their temporal dynamics (Carrasco, 2011): i.e., exogenous covert attention is “transient,” while endogenous covert attention can be “sustained” in time. Notably, people can voluntarily sustain their attention in space as long as it is needed by a given task and this voluntary deployment of attention takes about 300 ms to occur. On the contrary, exogenous attention has a faster time course than the endogenous counterpart, i.e., rises and decays quickly and peaks about 100 ms after stimulus onset (Hein et al., 2006; Ling and Carrasco, 2006; Busse et al., 2008). In the current study we will be addressing endogenous covert attention.

Electroencephalography (EEG) and magnetoencephalography (MEG) studies have explored the mechanisms underlying endogenous covert attention showing that the orienting of covert attention is reflected by the N2pc ERP component (observed about 200 ms after stimulus onset on contralateral posterior-occipital electrodes such as PO7, PO8, P7, and P8; Luck and Hillyard, 1994; Eimer, 1996). Other MEG and EEG studies have focused on oscillatory brain activity, showing that a change in alpha band power recorded from parieto-occipital channels is associated with changes in the orientation of covert spatial attention (Gould et al., 2011; Ikkai et al., 2016). In particular, alpha power decreases on the parieto-occipital channels that are contralateral to where attention is allocated.

The current study aimed at investigating whether the time course of the deployment of covert spatial attention leading up to the observer’s perceptual decision can be decoded from alpha power and raw activity traces, using multivariate pattern classification analyses (MVPA). Measuring these two EEG signals provides the additional advantage of being able to compare whether the raw activity trace and alpha power reflect the same or distinct features of attentional selection by looking at whether they decode attention in the same way prior to the observer’s perceptual response.

To date, studies have shown that both lateralized alpha suppression and ERP components such as the N2pc can be triggered by an attended visual target (Ekanayake et al., 2018). For instance, in a recent study the locus of focal attention was decoded from the N2pc component emerging after stimulus onset, using MVPA (Thiery et al., 2016; Fahrenfort et al., 2017). However, only alpha-band power has been used to provide a *continuous* temporal prediction of the orientation of attention before stimulus onset or response. Notably, several studies have shown that suppression of alpha-band power over contralateral channels can provide a reliable and continuous prediction of the orientation of attention before the onset of the attended target (e.g., Worden et al., 2000; Gould et al., 2011; Foster et al., 2017; Bae and Luck, 2018; Wen et al., 2019). Accordingly, spatial attention shifts can be reliably and continuously decoded from alpha-band activity thus allowing for the control of external devices via brain-computer interfaces (Bahramisharif et al., 2010; Treder et al., 2011). However, it is not yet known whether raw EEG signals can also provide a continuous prediction of the deployment of spatial attention before the presentation of the attended target. Hence, in the present study we investigated whether after the presentation of an attentional cue (cueing where the target will appear) and before observers’ perceptual responses, raw activity can reliably and continuously predicts the orientation of spatial attention. The same decoding technique was applied to alpha power and the results of the two brain signals were compared. One of the advantages of decoding attention from raw EEG signal is that it can provide an indication of where attention is located with high temporal resolution, thus supporting the fast interpretation of brain signals required in BCI. In contrast, alpha-band activity is limited to a resolution of about 125 ms (for 8 Hz alpha frequency) which may entail a loss of temporal information.

Participants viewed two Random Dot Displays (RDDs), one to the left and the other to the right of a central fixation point. An arrow was briefly presented at the center of the screen and served as a 100% predictive cue indicating the RDD in which coherent dot motion would be presented. Importantly, coherent motion would gradually appear (i.e., frame by frame more and more dots would move coherently in one of two directions, upward or downward) and participants were instructed to indicate the direction (up or down) of the coherently moving dots as soon as they detected it. Participants were also presented with neutral trials in which the arrow was replaced by a neutral cue conveying no information regarding the location of the upcoming dot motion. During the experiment, we recorded participants’ EEG activity and eye position using an eye-tracker. Eye tracking data allowed us to check that participants maintained central fixation throughout a trial and to assess the presence of possible oculomotor artifacts that could have contaminated EEG signals (i.e., microsaccades Engbert and Kliegl, 2003).

Using multivariate pattern classification analysis we assessed whether both alpha power and raw channel activity recorded during the cue-response interval could predict the orientation and temporal dynamics of covert attention. Our results corroborate the notion that alpha power and raw activity traces reflect different aspects of spatial attention, with the first associated with the orientation of covert attention in space (cf. Gould et al., 2011) and the second reflecting the modulation of sensory processing by attention (cf. Kiss et al., 2008).

## Materials and Methods

### Participants

Fourteen participants (four male, average age = 24.57, SD = 4.34) were recruited for a reimbursement of 15 €. One participant was removed from the sample due to very noisy EEG signals. The choice of this sample size was based on a similar experiment that we conducted previously and supported by robust effect size and power values in our analysis here (see Supplementary Materials). All participants had normal or corrected-to-normal vision, and were naïve to the hypothesis under investigation. They all gave written and informed consent before participating in the experiment. This study was conducted in agreement with the requirements of the Helsinki convention and approved by the local ethics committee of Université Paris Descartes.

### Apparatus

Stimuli were presented on a CRT Sony GDM-C520 (100 Hz refresh rate) with a resolution of 1024 × 768 pixels. Stimulus presentation and data collection were performed using MATLAB with the Psychophysics Toolbox (Pelli, 1997) and Eyelink Toolbox (Cornelissen et al., 2002) extensions. EEG data were recorded with 64 Ag/AgC1 electrodes mounted on an elastic cap and amplified by an ActiCHamp amplifier (Brain Product). The sampling rate of the EEG recording was set to 500 Hz. Electrodes were arranged according to the international 10–20 systems. Two of the electrodes (FT9 and FT10) were used to record horizontal eye movements (left and right HEOG, respectively), and two other electrodes (TP9 and TP10) were placed on the mastoids (M1 and M2, respectively). The right mastoid (M2) was used as online reference. Viewing was binocular and movements of one eye were monitored with an EyeLink 1000 (SR Research, Mississauga, ON, Canada) at 1000 Hz sampling rate. Head movements were restrained with a chinrest located 51 cm from the screen.

### Stimuli

Participants were presented with two Random Dot Displays (RDDs) one to the left and the other to the right of a central fixation point (a white empty circle of 0.3° visual angle diameter), with an eccentricity of 6° (from fixation to the center of the RDDs). The RDDs consisted of 80 white dots of a diameter of 3 pixels each (0.132°) displayed on a mid-gray background within a circular aperture of 4° diameter. When random dot motion was displayed, the dots moved in all directions with a speed of 1.5°/s. When coherent motion was displayed a proportion of dots (see section “Procedure” below) moved coherently either upward or downward with a speed of 3°/s. We used an increased speed for the coherently moving dots to facilitate the discrimination of upward and downward motion. Indeed, in pilot experiments we noticed that for some participants it was very hard to dissociate upward and downward directions with dots moving at 1.5°/s even when all dots moved in one of these directions (e.g., some participants systematically reported the opposite of what was displayed). The dot lifetime was set to 140 ms (14 frames at 100 Hz frame rate) after which it was erased and then displayed in another location within the circular aperture.

#### Establishing Individual Motion Discrimination Thresholds

Before starting the main experiment, we established participants’ motion discrimination thresholds. In this preliminary experiment, at the beginning of each trial, participants were presented with a fixation point. Participants were required to keep their eyes on the fixation point throughout the trial. 1000 ms after the onset of the fixation two Random Dot Displays (RDDs) were presented one on the left and the other on the right of fixation. The two displays were presented on the screen for 1500 ms. A proportion of dots of one of the RDDs (i.e., left or the right display) moved coherently either upward or downward, while the dots in the other display moved incoherently. The location (left or right) and direction (upward or downward) of the coherently moving dots was selected randomly. When the dot displays disappeared from the screen, participants were required to report the direction of the coherent motion (up or down) by pressing one of two designated keys always with their right hand, irrespective of the location of the motion (left or right). No time limit was given to participants’ response. If an eye movement or a blink occurred while the stimuli were displayed the trial was interrupted and a feedback appeared on the screen “please fixate” for 800 ms followed by a blank of 500 ms. The trial restarted after this blank screen.

Three interleaved staircases controlled the percentage of dots moving coherently. Each staircase started with a different percentage of dots moving coherently, i.e., 90, 80, and 70% of dots, respectively. If the participant’s response was correct (incorrect) the percentage of dots moving coherently (upward or downward) decreased (increased) in the subsequent trial. The size of this increment/decrement was controlled by an accelerated stochastic approximation algorithm (Kesten, 1958) set to converge at the percentage of dots moving coherently that supported 0.75 correct responses. Each staircase stopped once the convergence level was reached. The convergence values obtained with the three staircases was averaged and used as the participant’s upward vs. downward discrimination threshold. This threshold was used for both left and right dot display. Note that discrimination threshold can vary for stimuli presented to the left or the right visual field. Accordingly, when using the same threshold for both sides, participants might exhibit different accuracies and response times for the left and right target trials in the main task. The decision of using the same threshold for both sides was based on the fact that no difference in correct responses and response times were observed in pilot experiments. Control analyses assessing the response times and correct responses for left and right target trials observed during the main task are reported below.

This preliminary experiment lasted about 20 min.

### Main Experiment

#### Procedure

**FIGURE 1:**
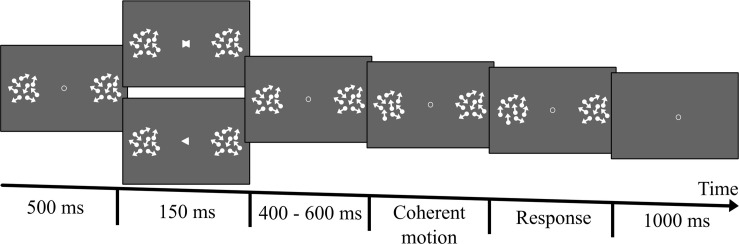
Illustration of one trial of the main experiment. A trial began with the presentation of a fixation point at the center of the screen. Two Random Dots Displays (RDDs) were then displayed only if participants kept their eyes on the fixation for at least 200 ms. More specifically, the verification of fixation was performed for a maximum time period of 1 s. If during that time period, participants did not fixate for at least 200 ms, the trial was interrupted and a calibration was proposed. A cue was presented 500 ms after the presentation of the RDDs. In the attention trials the cue was an arrow pointing either to the left or the right RDD and indicating where a coherent motion (upward or downward) would appear (left or right). The cue was displayed on the screen for 150 ms and was 100% valid. Cue orientation was selected randomly and equiprobably. 400, 500, or 600 ms after the onset of the arrow (each delay was selected on a random basis and equally often), the dots of the RDD indicated by the cue started to move coherently either upward or downward. The number of dots moving coherently increased linearly for 3000 ms until reaching a proportion equal to three times the threshold obtained in the preliminary experiment. At the end of the 3000 ms the number of dots moving coherently stopped increasing and coherent motion was displayed for additional 200 ms. Then, the dots disappeared and participants were required to not blink/move their eyes for additional 1000 ms. Participants were instructed to continuously monitor the cued RDD without moving their eyes, and to report the direction of coherent motion (upward or downward) as soon as they perceived it, by pressing one of two designated keys (the upward and downward arrows of the keyboard) with their right-hand index finger. The neutral trials were exactly the same, except that the arrows were replaced by a neutral cue created by the superposition of the two arrows. Attention and neutral trials were randomized and were presented equally often.

Once individual motion discrimination thresholds were established, participants completed the main experiment, which consisted of two experimental conditions (*Attention, Neutral*) during which their EEG brain activity was recorded (Figure 1 illustrates one trial of the main experiment). Attention and neutral trials were equiprobable and were randomized within the task. In the *attention* condition, participants were presented with a fixation point and two Random Dots Displays (RDDs). They were asked to keep their eyes on the fixation throughout the trial. An arrow (i.e., a white equilateral triangle of 0.5° height), pointing to the left or the right display, was presented at the center of the screen 500 ms after the onset of the trial for a duration of 150 ms. The arrow served as a cue indicating the side on which the dots would move coherently. The cue was 100% valid, but its orientation (left or right) was selected randomly and equiprobably.

In this experiment, contrary to the preliminary study, the number of dots moving coherently increased linearly over time. Notably, 400, 500, or 600 ms after the onset of the arrow (each delay was selected on a random basis and equally often), some of the dots of the RDD indicated by the cue started to move either upward or downward. More specifically, the upward/downward motion began with 1 of the dots moving in the specified direction. The number of dots moving coherently then increased linearly for 3000 ms until reaching a proportion equal to three times the threshold obtained in the preliminary experiment. For instance, if the threshold of a given participant was 0.2 of the dots (20%) moving coherently, then the proportion of coherently moving dots incremented until reaching 0.6 (i.e., 3 × 0.2). The size of the increment at each frame depended on the participant’s discrimination threshold and on the onset time of the coherent motion (400, 500, or 600 ms after the cue). Importantly, the dots of the uncued RDD moved incoherently throughout the trial. At the end of the 3000 ms the number of dots moving coherently stopped increasing and coherent motion was displayed for additional 200 ms. Then, dots disappeared and participants were required to not blink/move their eyes for additional 1000 ms. This time period was introduced to calculate the EEG baseline.

Participants were instructed to continuously monitor the cued RDD without moving their eyes, and to report the direction of coherent motion (upward or downward) as soon as they detected it, by pressing one of two designated keys (the upward and downward arrows of the keyboard) with their right-hand index finger. While the stimuli were displayed on the screen, participants were instructed to not move their eyes or blink. In the case of an eye blink or a saccade the trial was interrupted and a new trial added at the end of the trial list. A saccade was considered to have been initiated when gaze direction moved 2° away (vertically or horizontally) from the center of fixation. Similarly, if the participant failed to respond by the end of the presentation of the coherent motion an error message was displayed (“*too late*”) and a new trial was added to the end of the trial list. At the end of each trial, participants had 1 s to blink. A break was introduced every 25 trials where the experimenter could also perform an eye-tracker calibration if required.

The *neutral* condition was the same as the attention condition except that the location (left or right) of the coherent motion was not cued. The arrows were replaced by a neutral cue created by the superposition of the two arrows. Thus, participants did not know where the coherent motion was going to be displayed (left or right RDD) and had to monitor both sides. The neutral condition allowed us to evaluate whether we successfully manipulated attention in the cued trials.

The experiment stopped as soon as participants completed 240 attention and neutral trials without errors [i.e., 2 (attention, neutral) × 2 (left, right target) × 120 trials]. In total, the main experiment lasted on average 2 h 30 min (this also included EEG preparation time, instructions and pauses).

### Data Analyses

#### EEG Preprocessing

Electroencephalography data were preprocessed using the EEGLAB toolbox (Delorme and Makeig, 2004) and Fieldtrip (Oostenveld et al., 2011). We re-referenced the data to average left and right mastoids and then filtered with a low (48 Hz) and high-pass (0.05 Hz) Butterworth non-causal filter. In this research we were interested in the time dynamics and latency of attention processes, we thus decided to use a non-causal filter to avoid phase delays and thus changes in peak latency that can occur with causal filters (Rousselet, 2012; Cohen, 2014; Luck, 2014). However, note that non-causal filters might also introduce distortion to the data by injecting artifacts in time points prior to true events. For instance, it has been shown that a non-causal high-pass filter with a cut-off frequency of 0.3 Hz or higher produced artifactual effects of opposite polarity preceding the true effect (Tanner et al., 2015). Accordingly, our high-pass and low-pass frequencies were carefully chosen based on the recommendations of Tanner et al. (2015). Moreover, we tested our filter settings on simulated data to make sure that no distortion were introduced to the EEG traces (see Supplementary Material).

Reaction times varied across trials, conditions and participants. In order to be able to compare our conditions we selected the time limit of our EEG epochs respecting the following constraints: (1) maximize the number of trials to analyze, and (2) have the longest possible time period prior to the response. Thus, we selected epochs 1500 ms long, time-locked to the onset of participants’ response (i.e., from −1500 to 0 ms).

Electroencephalography epochs were corrected with a 500 ms baseline time-locked to the onset of the response i.e., from 500 to 1000 ms after the response. This linear baseline correction was used for the classification of raw channel traces. However, for time-frequency analyses we used a 200 ms linear baseline correction – from 800 to 1000 ms after the response – during preprocessing, and then a 500 ms time-frequency baseline – from 200 to 700 ms after participants’ response – to calculate average frequency power. Epochs containing amplitudes greater than 100 μV or less than −100 μV were marked as potential artifacts and then removed after confirmation through visual inspection. This led to the removal of 10 and 9% of trials in the neutral and the attention condition, respectively (i.e., an average of 21 and 19 trials in the neutral and the attention condition, respectively). Noisy electrodes were identified through visual inspection. Notably, electrodes exhibiting sudden and large changes of amplitude, or electrodes that were responsible for more than 20% of the trials to be removed during artifact rejection, or finally electrodes exhibiting large amplitude activity at high frequencies. The electrodes that were noisy for most of the participants and conditions were removed from classification analyses (T7, T8, TP7, TP8, Fp1, Fp2, FT7, FT8, AF7, AF8). The channels lHEOG, rHEOG were also removed from classification analyses. For some participants, we interpolated across electrodes that were noisy and that were located in the regions that have often been associated with attentional processes (i.e., central, parietal, and occipital regions). Specifically, the electrodes O1, FC1, and PO3 were interpolated for participants 4, 6, and 7, respectively.

For the classification analyses of raw EEG activity (see below) we averaged the time-points of EEG epochs corresponding to 10, 50, and 100 ms time windows. This was performed to assess whether classification accuracy based on raw activity would improve with increasingly larger time windows.

#### Time-Frequency Analyses

Trial-by-trial time-frequency activity for alpha oscillations was calculated using two separate methods. Notably, we performed a Hilbert transform on band-pass filtered data [with 8 and 13 Hz high and low-pass filters, respectively, as described in Foster et al. (2017)] and a Wavelet transform. Since both methods gave very similar results we present here only the Wavelet transform. The Wavelet transform provides a good compromise between time and frequency resolution (Tallon-Baudry and Bertrand, 1999). More specifically, trial-by-trial time-frequency activity was obtained for each participant, condition and electrode by applying Morlet wavelets, with linearly increasing cycles, to successive and overlapping time windows (Delorme and Makeig, 2004) of 478 ms. The Morlet wavelet for this time window covers three cycles of the lowest frequency (7 Hz) and reached fifteen cycles for the highest frequency (30 Hz). This analysis gave the power for frequencies ranging from 7 to 30 Hz within an epoch. Because the epoch started at −1500 ms, and the width of the moving window was 478 ms, this provided spectral estimates from −1262 to 760 ms. This is because spectral estimates can be obtained only at the center of the window. Trial-by-trial alpha power used for the classification analyses was calculated by squaring the absolute values of the complex numbers obtained with the Wavelet transform. In addition, a similar analysis was conducted on epochs time-locked to the onset of the neutral/attentional cue rather than to the onset of response. This analysis can be found in the Supplementary Material.

#### Multivariate Classification Analyses

Single trials were used to train a Linear Discriminant Analyses classifier (LDA; Carlson et al., 2003) using the available electrodes. In addition to LDA, other classifiers types were evaluated on a subset of the data, including a support vector machine and a multi-layer perceptron with a large range of layers/neurons. Because all types of classifiers led to similar results, only the LDA results are presented here.

Our main interest was to evaluate whether we could dissociate and predict from time-point-by-time-point EEG signals (raw activity and alpha power) the left/right direction of participants’ covert attention leading up to their perceptual judgments. Thus, separate LDA classifiers were trained and tested for each time-point in the EEG epoch to dissociate left and right target trials in both the attention and the neutral condition.

Our classification procedure implemented a Monte Carlo cross-validation method (Dubitzky et al., 2007). Notably, each classifier was trained on 90% of the available dataset and tested on each of the remaining trials. This procedure was repeated 200 times. Each time a random 90% of trials was used as training set and the rest as test set.

Classification accuracy was estimated with a Receiver Operating Characteristic (ROC) curve analysis and was summarized by the Area Under the Curve (AUC). The ROC curve presents the hit rate (the proportion of trials A classified as A) as a function of the false alarm rate (the proportion of trials B classified as A). It provides a less biased measure of classification accuracy when compared to the proportion of correct classification. Indeed, biased measures of classification accuracy can emerge, for instance, when two classes have unequal numbers of observations. In this case a classifier might wrongly categorize trials more frequently as those belonging to the category with the higher number of observations, thus artifactually boosting correct classifications. In the current experiment, after artifact rejection, some participants ended up with a slight imbalance in the number of trials in the left and right target samples of the attention and neutral condition. Hence even though computationally more demanding, we preferred to calculate AUC values. A diagonal ROC curve which corresponds to an AUC of 50% reflects a situation in which hit and false alarm rates are equal, showing that the classifier is at chance. In contrast, an AUC of 100% indicates perfect performance with 100% hit and no false alarm. Accordingly, unlike the proportion of correct classification, AUC values provide a less biased measure of decoding accuracy compared to the proportion of correct classification. Note that in our case the difference in number of trials for the left and right target trials was very small. Notably, the highest difference between the two classes was five trials. The mean AUC value of these 200 repetitions within each subject and time-point was taken as the classifier accuracy for that participant and that time-point. To evaluate whether the decoding accuracy was significantly above chance level (50%), we performed a series permutation tests as described below (a description of these tests on (M)EEG data can also be found in Maris and Oostenveld, 2007). In addition to permutation tests, other non-parametric statistical approaches were used to analyses decoding accuracy. For instance, we used signed rank tests comparing directly AUC values with chance level. Because all types of analyses led to similar results, only the permutation tests are presented here.

Firstly, individuals’ mean decoding accuracy (AUC) for each time-point was compared against chance level (50%). Notably, we subtracted 50% from the participants’ average AUC values observed at each time-point, and then performed a series of Wilcoxon signed rank tests on the resulting values. From these signed rank tests we estimated *z-*statistics. We also performed this analysis using paired Student *t*-tests, and the results were very similar.

Subsequently, we repeated these steps 1000 times. Each time we randomized the left and right trial labels of our training set. This way we were able to obtain 1000 AUC values for each time-point and participant, and consequently 1000 *z-*statistics representing the distribution of test statistic under the null hypothesis (i.e., no difference between left and right target trials)^1^. The same analyses were performed by randomizing both the labels of the training and of the test set. This led to very similar results to those reported below.

Finally, to calculate a *p*-value for each time-point we computed the proportion of random samples that resulted in a higher *z-*statistics than the one we obtained with the real sample (statistical significance was set to *p* < 0.05). *P*-values were corrected using False Discovery Rate (FDR) procedure as described in Benjamini and Hochberg (1995), a procedure designed to control the expected proportion of “rejected null hypotheses” that are false incorrect rejections and that can be used for both independent and dependent tests. As with other correction methods, FDR provides a critical *p*-value below which a statistical test can be considered as significant. The critical *p*-value is obtained based on the number of tests performed and the actual *p*-values obtained in the test (see Benjamini and Hochberg, 1995 for more details). Critical *p*-value is provided together with the results reported below.

These classification and statistical procedures were applied to both raw EEG activity, the power observed for alpha band frequencies (8–13 Hz) and eye-position data (see below for more details).

#### Eye Movement Analysis

Eye-movement data were recorded and analyzed to control for potential oculomotor artifact that might have contaminated EEG data. For offline analyses, eye movement samples were smoothed with SR Research’s proprietary algorithms.

As for the EEG data, we segmented eye-gaze data into 1500 ms long segments (i.e., from −1500 to 0 ms) time locked to the onset of participants’ response. Firstly, we removed from the eye-movement data the corresponding trials marked as containing artifacts in the EEG data. This was performed because we were interested in eye-movement data principally to control for any possible oculomotor artifacts in the EEG data. From the remaining segments we identified microsaccades using the algorithm described in Engbert and Kliegl (Engbert and Kliegl, 2003).

We also analyzed whether the orientation of attention could be simply predicted by participants’ eye position prior to their response. The procedure we used to decode left and right target trials from eye-position data was exactly the same as the one described for the EEG signals. Moreover, as for the raw EEG activity, we averaged the time-points of eye-position data in 10 ms successive time windows.

#### Behavioral Data

We measured average reaction times defined as the response latency from the beginning of the coherent motion, and proportion of correct responses (i.e., correct identification of the coherent motion direction) for each participant and condition (attention and neutral). Their comparison allowed us to assess whether our cues successfully manipulated covert spatial attention. In particular, we expected faster reaction times (RTs) in the attention compared to the neutral trials.

Furthermore, we also investigated whether reaction times, number of responses and proportion of correct responses varied between left and right target trials both in the attention and the neutral condition. These analyses were performed in order to control for any potential lateralized confound that could contaminate EEG signals. The results of these analyses are reported in the Supplementary Material.

## Results

### Behavioral Data

The average proportion of dots moving coherently required to achieve a discrimination performance of ∼75% correct responses in the preliminary experiment was 0.48 (SD = 0.15). Coherence thresholds are relatively larger than previously reported (e.g., Cornelissen et al., 1995: 1500 ms display, 50 frames lifetime, 2.5°/s; Burr and Santoro, 2001: variable duration, 3 frames lifetime, 4.7°/s; de Bruyn and Orban, 1988: variable durations, speeds, contrasts). This might be due to a combination of the life-time (14 frames and 100 Hz refresh rate), motion speed (3°/s) and stimulus eccentricity used in the current study.

In the behavioral data of the main experiment, we first evaluated whether the cueing procedure successfully manipulated spatial attention. A two-sample Wilcoxon signed rank test showed no difference in the proportion of correct responses between neutral (M = 0.89; SD = 0.05) and attention condition (M = 0.90; SD = 0.06), *p* > 0.5. The same test on RTs showed faster responses in the attention (M = 1.822 s; SD = 0.272 s) compared to the neutral condition (M = 1.983 s; SD = 0.254 s), *p* < 0.001. These reaction times corresponded to an average percentage of 51% of dots moving coherently in the attention condition and 56% in the neutral condition. This confirms that the cueing procedure we adopted successfully manipulated spatial attention: participants required less time in the attention condition to achieve the same level of performance as in the neutral condition.

#### Raw Activity Traces

**FIGURE 2:**
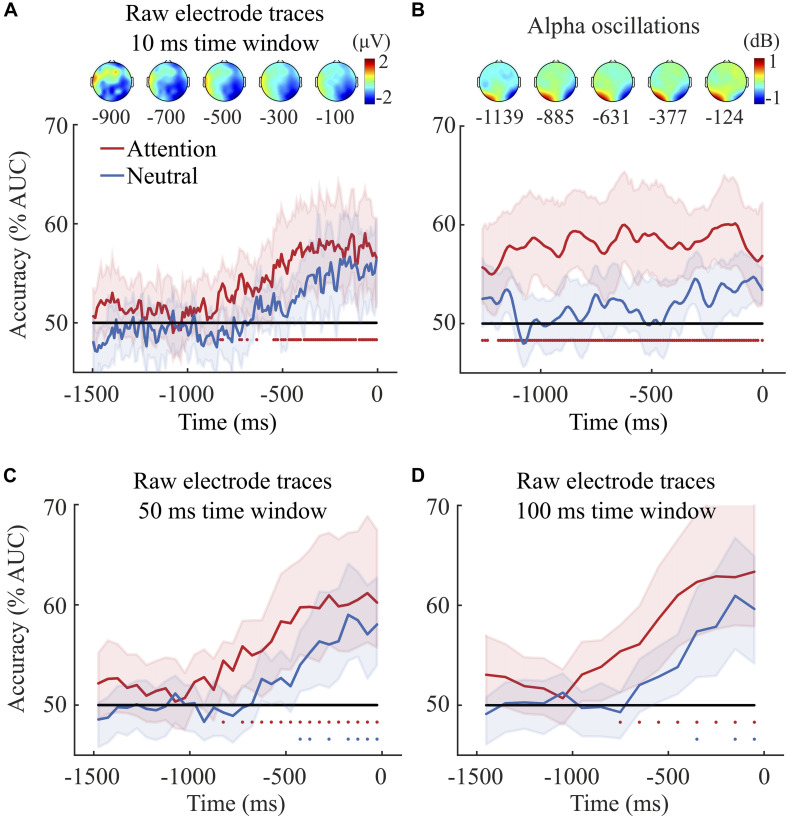
**(A)** Decoding accuracy based on raw activity traces (10 ms time window classifier). The classifier was trained to discriminate left from right target trials as a function of time. 0 ms indicates the onset of participants’ response for both the attention (in red) and the neutral condition (in blue) for raw channel traces. The classifier could dissociate left from right target trials only when target location was cued. Classification accuracy (AUC) was significantly above chance from about 826 ms before the onset of participants’ response. The red/blue dots under the lines indicate significant difference from chance tested via permutation tests. At 826 ms before the onset of participants’ response, about 28.75% of the dots were moving coherently. Note that participants’ average discrimination threshold was 48.15%. Since the average response time was 1819 and 1985 ms in the attention and neutral conditions, respectively, the coherent motion started on average prior to the beginning of the EEG segments shown here^2^. The percentage of dots moving coherently at **–**1500 and 0 ms was on average 9.65 and 52.31% in the attention condition, 14.13 and 56.92% in the neutral condition, respectively. The scalp distribution depicts the difference in brain activity between left and right target trials in the attention condition at five different 200 ms time windows prior to the onset of participants’ response with the midpoint of the window indicated under each on the graph. **(B)** Decoding accuracy for alpha power (8–13 Hz). The classifier could dissociate left from right target trials only when target location was cued. Classification accuracy (AUC) was significantly above chance from the beginning of the epoch, i.e., 1262 ms before the onset of participants’ response. Scalp topographies depict the distribution of alpha power (Event-Related Spectral Perturbations - ERSP) observed for left minus right target trials in the attention condition, at five different 200 ms time windows with the midpoint of each indicated underneath. Note that the average amplitude of alpha oscillation at each individual frequency was corrected with a 500 ms time-frequency baseline - from 200 to 700 ms after the onset of participants’ response. **(C,D)** The two bottom graphs depict the decoding accuracy (AUC) for the 50 and 100 ms time window brain potential classifiers. Classification accuracy for the 50 ms classifier was significantly above chance from 826 ms and 351 ms before the onset of participants’ response in the attention and the neutral condition, respectively. Importantly, at these time points, about 28.75% and 46.96% of the dots were moving coherently either upward or downward in the attention and neutral condition, respectively. Similar results were observed in the 100 ms time window classifier. Shaded areas represent bootstrapped confidence intervals.

We trained and tested separate classifiers to dissociate left from right target trials in the attention and in the neutral condition. Figure 2A shows the evolution of classification performance (AUC) over time for both conditions. The significance of decoding accuracy was calculated using permutation tests and *p*-values were corrected using FDR (see section “Data Analyses”). The results of the 10 ms raw activity trace classifier show that only in the attention condition could the classifier dissociate left from right target trials starting at about 826 ms prior to participants’ response (a total of 150 *p*-values, one for each time-point, were obtained with the permutation test, the critical *p*-value for significance after FDR correction was *p* ≤ 0.018). The absence of significant classification in the neutral condition indicates that decoding accuracy was not driven solely by the mere presentation of the coherent motion. A further non-parametric test corroborated this last point. First, we averaged the decoding accuracy (AUC values) across time-points for each condition (attention and neutral) and participants. Then, we used a non-parametric Wilcoxon signed-rank test to compare the averaged AUC values observed in the attention and the neutral conditions. The test showed that decoding accuracy was higher in the attention compared to the neutral condition (*p* = 0.039), thus confirming that in the attention condition we were not simply decoding the presentation of coherent motion but rather endogenous attention mechanisms.

Scalp topographies presented in Figure 2A show the brain activity observed in the left target trials minus the activity in the right target trials of the attention condition. The activity recorded by central, posterior and occipital electrodes differed the most between left and right target trials. A one-sample Wilcoxon signed rank test on linear coefficients (we averaged linear coefficients observed between −800 and 0 ms) confirmed this point showing that classification accuracy was mostly driven by central-posterior (CPz, CP4, CP6), posterior (Pz, P2, P4, P6, P8), posterior-occipital (POz, PO4, PO8) and occipital (Oz, O2) electrodes.

Interestingly, increasing the width of the window to 50 and 100 ms time improved classification accuracy in both the attention and the neutral condition. More specifically, we averaged the EEG activity for time points corresponding to 50 and 100 ms wide successive time windows. Indeed, in the attention condition, the 50 ms classifier could decode left and right target trials above chance from −826 ms to participants’ response (a total of 30 *p*-values, one for each time-point, were obtained with the permutation test, the critical *p*-value for significance after FDR correction was *p* ≤ 0.025). The highest AUC value with the 50 ms classifier was 61%, compared to 59% accuracy with the 10 ms classifier. Similarly, the 100 ms attention classifier could decode left and right target trials above chance from −751 ms to participants’ response with a maximum AUC value of 63% (a total of 15 *p*-values, one for each time-point, were obtained with the permutation test, the critical *p*-value for significance after correction was *p* ≤ 0.018).

We could also decode left versus right target trials above chance in the neutral condition from about 351 ms before participants’ response with the 50 ms time window classifier (a total of 30 *p*-values, one for each time-point, were obtained with the permutation test, the critical *p*-value for significance after correction was *p* ≤ 0.01). We could decode left and right target trials from about −426 ms before participants’ response with the 100 ms time window classifier (a total of 15 *p*-values, one for each time-point, were obtained with the permutation test, the critical *p*-value for significance after correction was *p* ≤ 0.01).

Further Wilcoxon signed-rank tests showed that for both the 50 and the 100 ms classifiers decoding accuracy was overall higher in the attention compared to the neutral condition, with the 100 ms classifier leading to the highest difference (Mean AUC value in the attention condition = 57%; Mean AUC value in the neutral condition = 52%, *p* = 0.006). This corroborates the notion that the presentation of coherent motion alone cannot explain the decoding accuracy observed in the attention trials. This observation was supported by further analyses aiming at classifying neutral versus attention trials using the 100 ms time window raw activity and alpha power (see Supplementary Material). The analyses showed that our classifiers could decode above chance whether a trial belonged to the attention or the neutral condition in several different time periods. This provides further evidence that what was decoded in the attention condition was not due to the mere presentation of the coherent motion but rather to attentional mechanisms (see section “General Discussion”).

#### Time-Frequency Data

As for the raw electrode activity, we trained and tested separate classifiers to dissociate left from right target trials in the attention and in the neutral condition using alpha power calculated by squaring the absolute values of the complex number obtained with a Wavelet transform. Figure 2Bshows the evolution of classification performance (AUC) over time for both the attention and the neutral condition. Performance was tested at each time-point by comparing the average classifier accuracy (AUC averaged across permutations for each participant) to chance level (50%). The results show that alpha power predicted the orientation of spatial attention from the beginning of the segment in the attention condition. Moreover, decoding accuracy was overall higher in the attention compared to the neutral condition (Mean AUC value in the attention condition = 57%; Mean AUC value in the neutral condition = 51%, *p* = 0.002). Scalp topographies depict the distribution of alpha power for left attention trials minus the right attention trials and show a change in alpha power over the parieto-occipital electrodes.

#### Comparison Between Alpha and Raw Activity

Firstly, we assessed whether there was a main effect of Signal (alpha vs. raw activity). Notably, for both the alpha and the raw activity classifiers, we averaged the AUC values across time for each classifier (Alpha, 10, 50, and 100 ms raw activity classifiers), condition (neutral and attention) and participant. We then compared the average AUC values with a series of Wilcoxon signed rank tests. The results showed that alpha power predicted the orientation of spatial attention overall better than the 10 ms raw activity classifier in the attention condition (signed rank = 14, *p* = 0.027; Mean AUC raw activity = 54%; Mean AUC alpha activity = 58%) but not in the neutral condition (*p* = 0.735; Mean AUC raw activity = 52%; Mean AUC alpha activity = 52%).

Subsequently, a series of paired Wilcoxon signed rank tests compared the time-point-by-time-point AUC values observed with the alpha classifier and the 10 ms raw activity classifier for both the neutral and the attention condition. A total of 125 tests were performed for each condition. FDR was used for correction to multiple comparisons (critical alpha for significance after FDR correction was *p* ≤ 0.0081). The analyses showed that alpha AUC values were higher than 10 ms raw activity AUC values between −1096 ms (±5 ms)^3^ and −612 ms (±5 ms) in the attention condition (*p* ≤ 0.0081). However, this difference disappeared for time-points superior to −612 ms (±5 ms), thus showing that raw activity led to the same prediction accuracy as alpha power for time-points closer to participant’s perceptual decisions. None of the comparisons in the neutral condition reached significance.

Further analyses explored whether alpha power would better predict the orientation of spatial attention also when compared to the 50 and 100 ms raw activity classifier accuracy. A series of Wilcoxon signed rank tests showed that the advantage of alpha we initially observed in the attention condition disappeared. Notably, no main effect of Signal (alpha vs. raw activity) was observed, neither for the 50 ms raw activity classifier (Mean AUC raw activity = 56%; Mean AUC alpha activity = 58%) nor for the 100 ms raw activity (Mean AUC raw activity = 57%; Mean AUC alpha activity = 58%). Similarly, the time-points-by-time-points comparisons showed that only at −1026 ms (±4 ms) were Alpha AUC values higher than the 50 ms raw activity AUC (*p* = 0.012; a total of 25 paired comparisons were performed, critical alpha after FDR correction was *p* ≤ 0.012). However, for all the other comparisons the AUC values observed with alpha did not differ from those obtained the time windowed raw activity. Consequently, the striking advantage of alpha in predicting spatial attention disappeared when compared to the 50 ms and the 100 ms time windowed raw activity AUC.

#### Eye-Tracking Data

The above analyses suggest that activity in parieto-occipital areas may be associated with the orientation of attention in space. However, specific eye movement signatures have also been linked to spatial attention, so it is possible that what we decoded was not attentional processes *per se*, but the activity guiding eye movements. To check whether this was the case, we ran several control analyses on eye position data.

**FIGURE 3:**
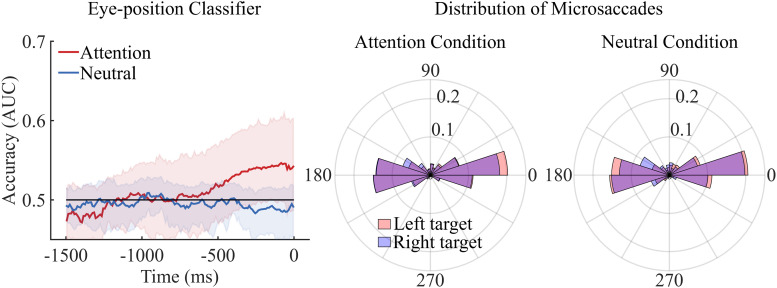
The left graph depicts decoding accuracy for the eye-gaze data. As for the raw EEG activity we averaged the time points corresponding to 10 ms in successive time windows. Neither the attention classifier nor the neutral classifier could decode significantly above chance level left versus right target trials. The same results were observed when time points were averaged in 50 and 100 ms successive time windows. Shaded areas represent bootstrapped confidence intervals. The central and right polar histograms depict the distribution of directions of microsaccades executed during left and right target trials for both the attention and the neutral condition. No difference was observed in the distribution of microsaccades between left and right target trials of the attention and neutral condition.

First, a classifier was trained and tested on eye position. Classifiers could not dissociate left from right target trials either in the attention condition or in the neutral condition. This means that eye position itself did not predict the orientation of spatial attention, and thus cannot explain, at least not entirely, the activity found in the EEG decoding analyses (see Figure 3, left graph).

Second, we assessed the correlation between eye position and brain activity recorded by fronto-lateral electrodes. We subtracted the brain activity recorded by the right frontal electrodes (rHEOG, Fp2, AF8, F8) from that recorded by the left frontal electrodes (lHEOG, Fp1, AF7, F7). We then calculated a time point-by-time point linear regression of average brain activity difference on the average horizontal component of eye position for each participant. Given that eye position was recorded at 1000 Hz and EEG at 500 Hz, we subsampled eye position data to the time points that were available in the EEG data. If participants performed eye-movements in the direction of the target and these movements drove the brain activity, we expected that the more leftward the horizontal component of eye-position, the more positive the left fronto-lateral EEG activity would be relative to right fronto-lateral. A one sample signed rank tests showed that the slope (M = −8.96, SD = 20.47) of the linear model was not significantly different from zero, signed rank = 69, *p* = 0.11. This corroborates the classification result presented above, showing that eye position did not reliably predict the allocation of spatial attention and that ultimately classification accuracy on raw channel activity could not be solely attributed to oculomotor factors.

Previous research showed that microsaccades, rather than eye-position data, show the strongest association to the orientation of spatial attention (Engbert and Kliegl, 2003). Accordingly, we performed further analyses evaluating whether microsaccades differed between left and right target trials and thus could potentially explain our decoding accuracy.

First, we computed the distribution of directions of microsaccades for each participant and condition. Our statistics were performed with MATLAB in combination with the circular statistics toolbox (Berens, 2009). We then calculated the mean, median and standard deviation of these distributions of directions and we performed a series of Wilcoxon signed rank tests to evaluate whether the mean/median/sd directions observe in the left and the right target trials differed in the attention and the neutral condition. No difference was observed between left and right target trials in the attention (*p* = 0.54) and the neutral condition (*p* = 0.45). Similarly, no difference was observed between the attention and neutral condition (*p* = 0.30).

Second, we pooled together observers’ distribution of microsaccade directions for left and right target trials in the neutral and attention condition. We then compared these distributions using a non-parametric test for circular data (i.e., the multi-sample test for equal median directions described in Berens, 2009). The analyses confirmed that the direction of microsaccades did not differ between left and right target trials in the attention condition or the neutral condition, *p* = 0.11 and *p* = 0.14, respectively (see Figure 3, central and right graph).

Third, in order to assess a possible contribution of microsaccades on decoding accuracy, we calculated a linear correlation between EEG decoding accuracy and the proportion of microsaccades performed in the direction of the dot display containing the coherent motion. Notably, we wanted to investigate whether the participants who performed more microsaccades in the direction of the coherent motion were also those who exhibited the higher EEG decoding accuracy. In particular, we assessed whether the proportion of saccades in the direction of the target correlated with the decoding accuracy observed at the last time-point of our 10 ms brain potential classifier. The Pearson correlation coefficient did not reach significance (*p* = 0.764). The same analyses were performed with the other classifiers leading to the same result.

Finally, we investigated whether the decoding accuracy based on eye-position data was correlated with the decoding accuracy based on the raw activity classifiers. Notably, we wanted to assess whether the participants’ with higher decoding accuracy from the eye-position data were also those who showed higher decoding accuracy with the 10 ms raw activity classifier. The Pearson correlation coefficient (value) was not significant (*p* = 0.47).

In sum, none of the analyses performed on eye-tracking data provided evidence that the orientation of spatial attention could be predicted by eye-gaze or eye-movements. Furthermore, no relationship between the proportion of microsaccades performed in the direction of the target and EEG decoding accuracy was observed. Accordingly, EEG decoding accuracy cannot be explained solely by eye-movements. Further control analyses investigating the relationship between EEG and eye-movement data are presented in the Supplementary Material.

#### Control Analyses

Further behavioral analyses investigated whether number of responses, reaction times and proportion of correct responses differed between left and right target trials in the attention and neutral condition. As we reported above, the same discrimination threshold was used for both left and right dot display. This could potentially represent a problem if the discrimination threshold is different for the left or the right visual field (Greenwood et al., 2017). Accordingly, when using the same threshold for both sides, participants might show different accuracies and reaction times in the main task for the left and right side. Consequently, it could be argued that decoding accuracy (see below) observed with raw signals could be due to difference in response time when coherent motion was presented on the left and right of fixation. A series of Wilcoxon signed rank tests showed no difference in reaction times between left (M = 1.83 s, SD = 0.32 s) and right target trials (M = 1.81 s, SD = 0.26 s.) trials in the attention condition (*p* = 0.45). Similarly, no difference was observed (*p* = 0.27) in the neutral condition (Left trials: M = 2.00 s, SD = 0.32 s; Right trials: M = 1.96 s, SD = 0.24 s).

In addition, no difference was observed in the proportion of correct responses for left and right target trials either in the attention condition (*p* = 0.11; Left trials: M = 0.92, SD = 0.06 s; Right trials: M = 0.88, SD = 0.09) or in the neutral condition (*p* = 0.84; Left trials: M = 0.90, SD = 0.05 s; Right trials: M = 0.89, SD = 0.08). The same results were observed when performing parametric statistics.

These analyses confirmed that decoding accuracy observed with raw EEG traces could not be simply attributed to biases in participants’ responses. Further analyses corroborated this point. We evaluated whether EEG decoding accuracy was correlated to the difference in response time between left and right target trials. Notably, we wanted to investigate whether the participants’ for whom the difference in response time for the left and right target trials was the highest would also exhibit higher decoding accuracy. Hence, firstly we calculated the absolute difference between the average response time observed in the left and right target trials for each participant, and we assessed whether this difference correlated with the classification accuracy observed at the last time point of our 10 ms raw activity classifier. The Pearson correlation coefficient did not reach significance (*p* = 0.76). Therefore, classification accuracy cannot be explained by response times. The same analyses were performed also with the other classifiers leading to the same result.

We also investigated whether participants performed more “upward” or “downward” responses when the coherent motion was presented to the left or to the right, to rule out the possibility that decoding accuracy was contaminated by participants’ judgments (up or down). In order to address this issue, we first calculated the ratio of upward and downward responses both when the coherent motion was presented to the left and when presented to the right for the attention and neutral conditions (i.e., we simply divided the number of upward responses by the number of downward responses for each target side and condition). Then, we performed a Wilcoxon signed rank test comparing the ratio of responses observed when the target was presented on the left and the right. The analyses show that the number of responses did not differ between left and right target trials either in the attention (*p* = 0.37; ratio or responses for left trials: M = 1.044, SD = 0.22; ratio or responses for right trials: M = 1.091, SD = 0.32) or in the neutral condition (*p* = 0.89; ratio or responses for left trials: M = 1.127, SD = 0.37; ratio or responses for right trials: M = 1.145, SD = 0.40). Consequently, no difference between the numbers of responses was observed. We are confident that decoding accuracy was not due to motor or more general response pattern of the participants.

## General Discussion

We decoded the temporal dynamics of covert spatial attention from both raw activity and alpha oscillations. We used a cueing task to manipulate participants’ allocation of attention in space with two conditions: a cued covert attention condition, and an uncued neutral condition. Coherent motion was presented gradually in either the left or the right random dot display and participants were required to report the direction (upward or downward) of coherent motion as accurately and quickly as possible. In the neutral condition, participants continuously monitored both left and right random dots displays, whereas in the attention condition, they monitored only the cued side.

We used multivariate pattern classification analysis applied to both raw activity and alpha power to decode and predict whether participants were attending to the right or the left Random Dot Display (RDD). Based on raw EEG traces, we decoded whether participants were attending to the left or right RDD starting about 826 ms before their response in the cued attention condition. In addition, scalp topography of both raw EEG traces and alpha power pointed to prominent activity from parieto-occipital electrodes that have often been linked to the spatial attention network (Jensen and Mazaheri, 2010; Gould et al., 2011; Thiery et al., 2016). The implication of a parieto-occipital network in the deployments of spatial attention has also been demonstrated by a recent fMRI study using real-time decoding (Battistoni et al., 2018).

Interestingly, when individual time-points of raw activity were averaged across successive time windows of 50 and 100 ms, decoding accuracy improved compared to the 10 ms time windows. In the attention condition, the highest accuracy rose from 59 to 63% while in the neutral condition, accuracy rose to reach significance (i.e., 61%). With the wider time windows, we could dissociate left from right target trials starting from 751 and 426 ms before the onset of participants’ response in the attention and the neutral condition, respectively (in the 100 ms time-window classifier). The decoding accuracy observed in the neutral condition with the larger time windows suggests that in that case we were decoding either the increase of coherent motion or the moment when attention was captured exogenously by the coherent motion.

Decoding accuracy also improved when LDA classifiers were trained on alpha power compared to the 10 ms raw activity classifier. Importantly, all classifiers in the attention conditions led to a higher decoding accuracy than those in the neutral conditions, suggesting that decoding accuracy in the cued trials was not merely due to the gradual increase of coherent motion but rather to endogenous attention processes. This observation was corroborated by a series of classifiers aiming at dissociating neutral from attention trials for both alpha power and 100 ms time window raw activity (see Supplementary Material).

Our result replicates previous findings suggesting that alpha band oscillations are reliable signals to decode the deployment of covert spatial attention (Worden et al., 2000; Gould et al., 2011; Samaha et al., 2016; Foster et al., 2017; Bae and Luck, 2018). For instance, a recent study of Foster et al. (2017) showed that alpha power could track the deployment of covert attention in time.

Importantly, we show that raw EEG activity can also predict the temporal dynamics of spatial attention after attentional cueing and before perceptual responses (see also Wen et al., 2019). Previous research was able to decode the orientation of spatial attention in a particular time window, that is using the N2pc component which occurs about 200 ms after stimulus onset (Fahrenfort et al., 2017). Our study provides evidence showing that instead, raw EEG activity can provide a continuous prediction of the orientation of spatial attention starting from the moment that the sensory signal, in our case the coherent dot motion, can be perceptually dissociated from noise.

Moreover, the time profile of the decoding accuracy obtained with alpha and raw channel activity suggested that these classifiers decode two distinct processes. Specifically, the classifier applied to alpha power could dissociate left and right attention trials over the pre-response interval with about the same accuracy throughout. This suggests that spatial attention was oriented to the left/right random dot display already at the beginning of the segment when coherent dot motion was not yet distinguishable from incoherent motion. Indeed, at the beginning of the segment an average of only 9.65% of dots moved coherently, while the participants’ average discrimination threshold was 48.15%. In contrast, the classifier applied to raw activity showed a gradual increase of accuracy with time from about 826 ms before participants’ perceptual responses and when an average of 28.75% of dots moved coherently. This suggests that this classifier might be decoding the application of attention to the sensory signal (i.e., the coherent dot motion), a process that can only begin once the signal can be more easily dissociated from noise. In other words, alpha power could be associated with the orientation of covert attention in space, whereas raw channel activity may reflect the influence of attention on perceptual processes. More specifically, the suppression of alpha power in contralateral electrodes would reflect preparatory processes that releases inhibition. Notably, cortical excitability would increase in the brain regions that process the upcoming target in the attended location, while the activity in the (ipsilateral) regions processing the unattended location would be inhibited (Jensen and Mazaheri, 2010; Haegens et al., 2011; Klimesch, 2012; Bae and Luck, 2018). However, like ERP components such as the N2pc, the classification based on raw activity might index the enhancement of target processing for the stimuli presented in the attended location (cf. Luck and Hillyard, 1994; Eimer, 1996; Kiss et al., 2008; Mazza and Caramazza, 2015). This interpretation seems to be corroborated by the time dynamics of decoding accuracy we observed for the 50 and 100 ms raw EEG classifier in the attention and neutral conditions where the increase of decoding accuracy coincides with the increase in coherent motion. Notably, decoding accuracy increased gradually in both neutral and attention condition with the increase of coherent motion, in a manner reminiscent of evidence accumulation (O’Connell et al., 2018). More specifically, decoding accuracy in these cases was significantly above chance from 826 to 351 ms before the onset of participants’ response in the attention and the neutral condition, respectively (for the 50 ms window classifier). Importantly, at these time points, about 28.75% and 46.96% of dots were on average moving coherently (upward or downward) in the attention and neutral condition, respectively. Interestingly, observers’ average discrimination threshold calculated during the preliminary experiment was 48.15% of dots moving coherently. Hence, the gradual increase of decoding accuracy above chance level in the neutral condition starts on average when coherent motion reaches observers’ discrimination threshold, while in attention condition the increase in classification accuracy starts earlier suggesting that attention improved observers’ discrimination threshold, thus corroborating the notion that attention enhances sensory processing.

Importantly, recent studies suggest that alpha oscillations do not only reflect preparatory mechanisms linked to the anticipatory deployment of attention in space, but they are also a marker for target processing in a similar way as components such as the N2pc. In line with this notion, a recent study showed that visual search targets triggered both an N2pc and a lateralized suppression of alpha-band activity (Bacigalupo and Luck, 2019). However, alpha oscillations and the N2pc exhibited different time dynamics. According to Bacigalupo and Luck (2019) their results indicate that lateralized alpha-band activity is involved in target processing and is not purely associated to anticipatory mechanisms, suggesting that alpha activity and N2pc reflect a related but separable mechanism of spatial attention. Accordingly, alpha oscillations may also have contributed the classification accuracy we observed with the raw EEG traces. Complementary analyses reported in the Supplementary Material aimed at investigating this point. Notably, we explored whether the decoding accuracy observed in the raw EEG classifier would persist also after removing alpha oscillations from raw EEG activity. The analyses showed a very similar pattern of results as the one observed with raw EEG classifiers, suggesting that alpha contributed but it cannot alone explain the classification accuracy observed with raw EEG traces.

Finally, in addition to alpha oscillations, also gamma band frequencies (>30 Hz) have been associated to the inhibition and selection of sensory information. For instance, Bonnefond and Jensen (2013) showed that, in a memory task, distracters elicited a lower gamma power and a higher alpha power compared to target stimuli. The authors suggested that while alpha oscillations would reflect anticipative modulation of excitability, gamma power would be linked to the interaction between this top-down modulation and the stimulus-driven activity. However, in the present study we were not able to decode the orientation of spatial attention from gamma power (see Supplementary Material).

Contrary to past research (Engbert and Kliegl, 2003; Yuval-Greenberg et al., 2014) we did not observe any difference in the direction of microsaccades when participants were attending the left or right dot display in our stimulus. This suggests that eye-movement artifacts cannot explain the decoding accuracy observed with our raw EEG traces, thus confirming that the activity we observed in parieto-occipital areas is related to attentional mechanisms rather than eye movements. However, our failure to replicate previous findings linking attention and microsaccades may simply be a result of our lack of statistical power. Microsaccades are infrequent (i.e., 1 or 2 microsaccades are observed every 1/2 s). Studies investigating microsaccades typically collect a very large number of trials (White and Rolfs, 2016). Given the length of our recording epochs of 1500 ms and the number of trials we analyzed (on average 110 trials per condition), we do not have the data density required to examine the link between microsaccades and attention. And, indeed, our study did not aim at investigating these differences. Eye-movements were recorded in order to control for any contamination of oculomotor artifacts on the EEG signals. In this respect, we believe that our results unambiguously show that the EEG decoding was not due to eye-movements.

The ability to decode brain signals linked to cognitive processes such as attention provides invaluable information about the *causal* relationship between brain and cognitive function (Poldrack, 2011) – a central goal of cognitive neuroscience. However, it can also guide the development of brain-computer interfaces (BCIs) that can help individuals control external devices using EEG (Lebedev and Nicolelis, 2006). This is particularly important for patients who have lost the ability to give motor responses. In this context, one of the advantages of EEG for the development of BCIs is its high temporal resolution allowing for fast interpretation of brain signals. Alpha-band activity can be tracked with resolution of about 125 ms (for 8 Hz alpha frequency), which may entail a significant loss of temporal information. In contrast, the raw EEG traces provide a reliable alternative to decode attention with little or no loss. In particular, when simply averaging the time-points of raw activity in 50 ms successive time windows the decoding accuracy matched that of alpha power while only sacrificing a small amount of temporal resolution. It is important to note, however, that our study suggests also that the decoding from alpha power and raw activity reflects difference processes of spatial attention. Hence, one signal cannot simply substitute for the other. The choice of the signals to use for BCI will need to be evaluated depending on the issue a given BCI is addressing (see Van and Jensen, 2009).

However, useful BCIs require higher classification accuracy than we have found here and several methods can be used to address this issue. For instance, Long Short-Term Memory (LSTM) neural networks can take into account the temporal dependency of EEG traces and may therefore improve classification accuracy (Hefron et al., 2017). In addition, instead of concentrating on individual time points, one could focus on classifier outputs observed within a predefined time window. Indeed, by averaging time points across time windows of 50 or 100 ms, we significantly improved classification accuracy. Alternately, other approaches might consist in calculating the votes for one class within a sliding time window of 100 ms, and then using Bayesian models to calculate a weighted sum over all votes (see Dietterich, 2000 for more details). The combination of this sliding window method and LSTM could potentially improve classification accuracy and generate more reliable decisions.

## Data Availability Statement

The raw data supporting the conclusion of this conclusions can be found here: https://osf.io/ge7jh/.

## Ethics Statement

This study was conducted in agreement with the requirements of the Helsinki convention and approved by the local ethics committee of Université Paris Descartes.

## Author Contributions

AD and PC conceived and designed the study. AD programmed the experiment and drafted the manuscript. AD analyzed the experiment with the contribution of AC-H-T and HH. All authors commented the drafted version. AD wrote the final version of this article with the contribution of all other authors.

## Conflict of Interest

The authors declare that the research was conducted in the absence of any commercial or financial relationships that could be construed as a potential conflict of interest.

## Footnotes

1 We have also directly compared the AUC values obtained with the actual data and the AUC values obtained with the randomized data. The results were similar (even stronger effects were observed in the attention condition). However, we preferred the approach reported here as it preserves inter-subject variability, while comparing directly the AUC values of the actual and the randomized data requires averaging the AUC values across participants and thus loosing inter-subject variability.

3 Note that the time-points of the 10 ms raw activity did not always match with the alpha time-points, and the largest difference between time points was 5 ms.
