# Supplementary material for: Decoding the Temporal Dynamics of Covert Spatial Attention Using Multivariate EEG Analysis: Contributions of Raw Amplitude and Alpha Power
Source: Front Hum Neurosci. 2020 Oct 9;14:570419. doi: 10.3389/fnhum.2020.570419 (PMC7586305; doi:10.3389/fnhum.2020.570419)

Supplementary Material

Decoding the temporal dynamics of covert spatial attention using multivariate EEG analysis: contributions of raw amplitude and alpha power

Andrea Desantis[1,2,3] Adrien Chan Hon Tong[1], Thérèse Collins[2], Hinze Hogendoorn[5,6]

and Patrick Cavanagh [2,6,7]

[1] Département Traitement de l'Information et Systèmes, ONERA, France

[2] Integrative Neuroscience and Cognition Center (UMR 8002), CNRS & Université de Paris, France

[3] Institut de Neurosciences de la Timone (UMR 7289), CNRS & Aix-Marseille Université, France

[4] Melbourne School of Psychological Sciences, The University of Melbourne, Melbourne, Australia

[5] Utrecht University, Helmholtz Institute, Department of Experimental Psychology, The Netherlands

[6] Department of Psychological and Brain Sciences, Dartmouth College, USA

[7] Department of Psychology, Glendon College, Toronto, Canada

Filter simulations and further analyses

The **left panel of Figure 1** depicts a simulated 10 Hz half cycle wave signal with 5 µV amplitude (in orange). The blue line reflects the simulated wave after applying the filter we used during pre-processing of the attention and neutral trials, i.e. a non-causal Butterworth filter with 0.05 Hz and 48 Hz high and low pass frequencies, respectively. As can be seen, the filter did not introduce any distortion to our data. In addition, we reanalysed part of our data without applying any filter at all. The **right panel of Figure 1** shows the classifier accuracy in dissociating left from right target trials from time-point-by-time-point raw EEG activity in the attention condition. The results are very similar to those we observed with the filtered data.

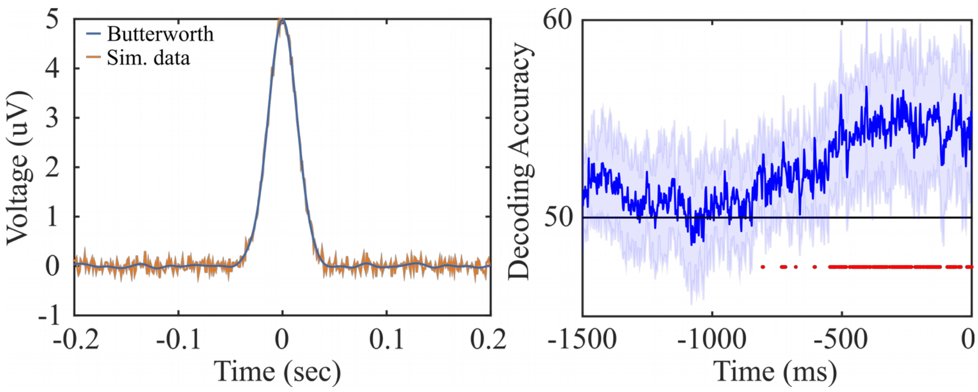

Figure 1

Effect size and achieved power

We calculated Cohen's d value for both the decoding accuracy of the 100 ms raw EEG classifier and the alpha classifier in the attention condition. For the raw EEG classifier, we averaged the decoding accuracy observed for the last 5 time points (where the effect was highly significant) for each participant and then calculated Cohen's d value on this average. This resulted in a very high effect size of 1.1314 and an achieved statistical power of 0.982. For the alpha classifier we averaged decoding accuracy observed across all time points for each participant, Cohen's d was then calculated on this average. This resulted in a very high effect size of 1.0846 and an achieved statistical power of 0.974.

Control analyses

Behavioural data. It is possible that participants performed more 'upward' or 'downward' responses, when coherent motion was presented to the left or to the right. Accordingly, it could be suggested that our classifiers are decoding participants' judgments (up or down) and not attention mechanisms. In order to address this issue, we firstly calculated the ratio of upward and downward responses both when the coherent motion was presented to the left and when presented to the right for both the attention and neutral condition (i.e. we simply divided the number of upward responses by the number of downward responses for each target side and condition). Then, we performed a Wilcoxon signed rank test comparing the upward/downward response ratio we observed when the target was presented on the left and on the right. The analyses showed that the number of responses did not differ between left and right target trial neither in the attention ($p = 0.37$; response ratio for left trials: M = 1.044, SD = 0.22; response ratio for right trials: M = 1.091, SD = 0.32) nor in the neutral condition ($p = 0.89$; response ratio for left trials: M = 1.127, SD = 0.37; responses ratio for right trials: M = 1.145, SD = 0.40). Consequently, no difference in the numbers of responses was observed (Table 1 shows the number of responses per condition). We are confident that decoding accuracy was not due to motor or more general response pattern of the participants.

Table 1. Number of responses per condition

	Left target<br>Response up	Left target<br>Response down	Right target<br>Response up	Right target<br>Response down
Attention	56	54.8	56.2	54.6
Neutral	56.8	52.9	56.5	53.3

Eye-movement data. In the following analyses we further investigated whether there was a difference in the distribution of microsaccades between our conditions. The first analyses explored whether participants performed more microsaccades in the direction of the RDD containing a coherent motion

with time. To evaluate this hypothesis we firstly determined for each participant and condition whether a given microsaccade was performed in the direction of the target (i.e. 1) or not (i.e. 0). We then run a linear regression model for each participant and condition (attention and neutral) to investigate whether the proportion of microsaccades in the direction of the target increased as a function of time. We estimated the slope of the model for each participant and condition. If the number of microsaccades increased as a function of time, we expected a positive slope. A series of Wilcoxon signed rank tests showed that the slopes did not differ from 0 (no slope) neither in the attention condition ($p = 0.07$ signed-rank = 19; mean = -0.00005), nor in the neutral condition ($p = 0.95$, signed-rank = 44; mean = -0.00002).

Further analyses investigated whether the participants that performed more microsaccades in the direction of the target in the attention condition (i.e. the participants with higher slope values of the linear models) also exhibited higher classification accuracy. The Pearson correlation coefficient was not significant ($p = 0.184$).

In conclusion, none of the analyses on eye-tracking data indicated that eye-position or eye-movements could predict the location of spatial attention. Consequently, the decoding accuracy observed with raw EEG signal cannot be attributed to any oculomotor artifacts.

Decoding neutral versus attention trials

Figure 2 depicts the decoding accuracy for a classifier trained to dissociate neutral and attention trials from the 100 ms time window raw activity (left graph) and alpha power (see classification procedure in the data analyses section). Blue points under the curve depict the time points in which the classifier could decode above chance whether a trial belonged to the attention or the neutral condition. Significant decoding accuracy was observed from 1351 ms to 351 ms before participants' response with the 100 ms time window classifier (critical p value after FDR correction $p \leq 0.031$). Statistical significance was determined with a permutation test (False Discovery Rate was used as a correction for multiple comparisons; see data analyses section). Decoding accuracy for the alpha oscillations exhibited several time points clusters above chance level: from -896 to -824 ms, from -500 to -306 ms, from -236 to -174 and from -64 to -32 ms before participants' response classifier (critical p value after FDR correction $p \leq 0.015$). These analyses corroborate the notion that we did not simply decode the mere presentation of coherent motion but rather attentional selection processes.

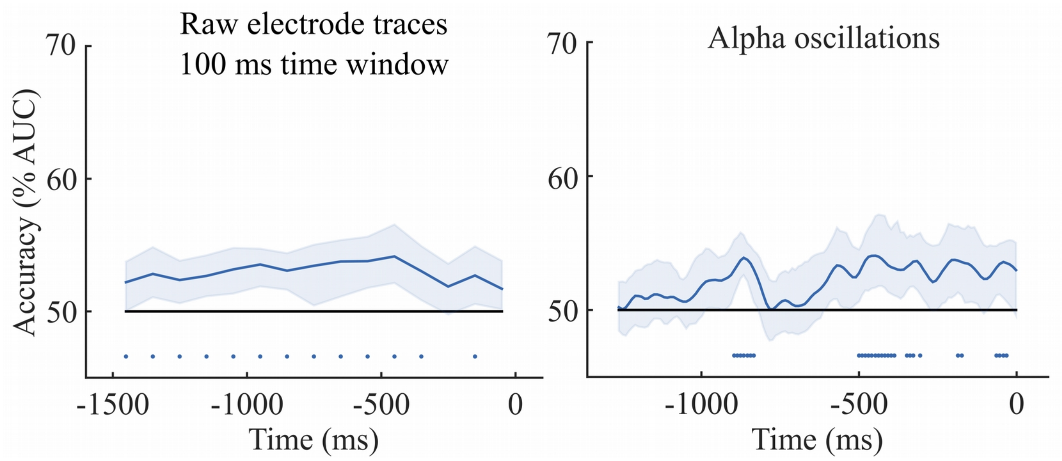

Figure 2

EEG analyses time-locked at cue onset

In the alpha power analyses reported in the manuscript we observed that we could decode the location of spatial attention from the beginning of the segment time-locked at response onset. If this decoding accuracy is related to endogenous attention rather than to artifactual activity unrelated to attentional processes, decoding accuracy should be at chance at cue onset and it should become significant after cue presentation. We performed this sanity check. Notably, new epochs were time-locked at cue onset [i.e. from -500 to 1500 ms]. We applied a 100 ms linear baseline correction [i.e. from -500 to -400 ms]. Epochs containing amplitudes greater than 100µV or less than -100µV were marked as potential artefact and then removed after confirmation through visual inspection. This led to the removal of a proportion of 0.05 and 0.06 trials in the attention and neutral condition respectively (i.e. an average of 13.1 and 14.7 trials in the attention and neutral condition, respectively). The electrodes O1, FC1 and PO3 were interpolated for participant 4, 6 and 7 respectively.

Subsequently, trial-by-trial time-frequency activity for alpha oscillations was calculated by applying a Wavelet transform (as for the analyses of the epochs time-locked to response onset). More specifically, trial-by-trial time-frequency activity was obtained for each participant, condition and electrode by applying Morlet wavelets, with linearly increasing cycles, to successive and overlapping time windows of 478 ms. Morlet wavelet started with three cycles for the lowest frequency (7 Hz) within the analysis window and reached fifteen cycles for the highest frequency (30 Hz). This analysis gave the power for frequencies ranging from 7 to 30 Hz within an epoch from −260 ms to 1260 ms.

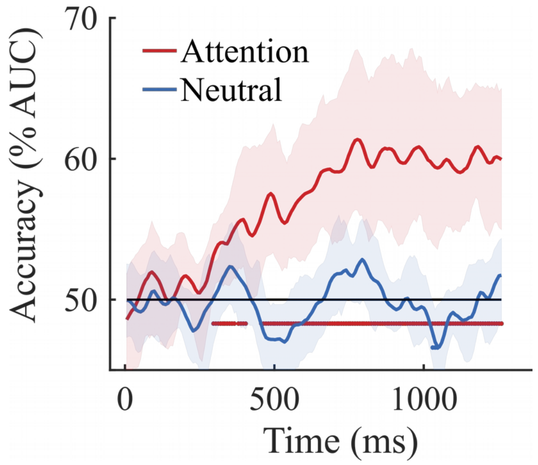

Figure 3

Trial-by-trial alpha power used for classification analyses was calculated by squaring the absolute values of the complex number obtained with the Wavelet transform. Furthermore, similarly to the analyses that were time-locked to response onset, the electrodes that were noisy for most of the participants and conditions were removed from classification analyses (T7, T8, TP7, TP8, Fp1, Fp2, FT7, FT8, AF7, AF8). The channels lHEOG, rHEOG were also removed from classification analyses.

Previous research showed that voluntary deployment of attention takes about 300 ms to occur (Carrasco, 2011). In line with previous observations, the results show (see Figure 3) that decoding accuracy was significant from about 304 ms after the onset of the cue in the attention condition. This support the analyses conducted on the epochs time-locked at response onset. Notably, participants orient their attention to the RDD that will display a coherent motion as soon as the cue is interpreted.

Contribution of gamma and alpha power on raw EEG classification

One might argue that the decoding accuracy observed with raw channel traces is in reality driven by alpha oscillations. Accordingly we performed further analyses to address this point. We investigated whether the decoding accuracy in the 10 ms raw EEG classifier was correlated with the decoding accuracy of the alpha classifier across participants in the attention condition. We performed a total of 125 Pearson's correlations, one for each time point matching between the two data sets. The figure below (Figure 4) depicts Person's correlation coefficient for each time points (blue line). The red points indicate the 23 time points with significant p-values **when no correction for multiple comparisons was applied** (p-values ranged from 0.002 to 0.049). Using the False Discovery Rate correction for multiple comparisons as described in the manuscript, none of the correlations was significant.

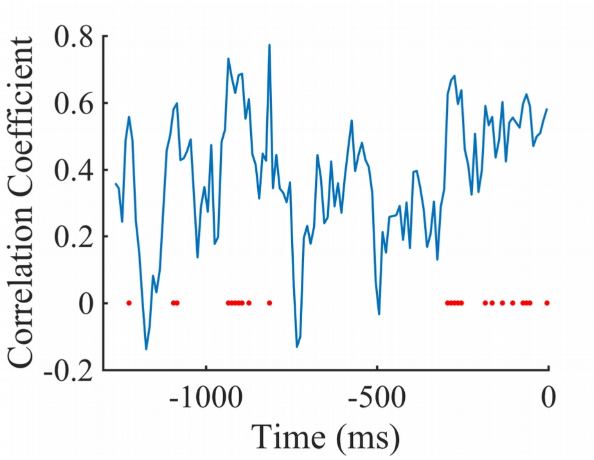

Figure 4

To further explore the contribution of alpha oscillations on the decoding accuracy observed with the raw EEG classifiers, we investigated whether the decoding accuracy observed in the raw EEG classifier would persist also after removing alpha oscillations from raw EEG activity. We performed a wavelet transform and extracted frequencies from 1 Hz to 48 Hz and we then reconstructed the original signal but without alpha frequencies (8 Hz – 13 Hz). The results showed a very similar pattern of results as the one observed with raw EEG classifiers, suggesting that alpha contributed but cannot alone explain the classification accuracy observed with raw EEG traces (see Figure 5 right graph).

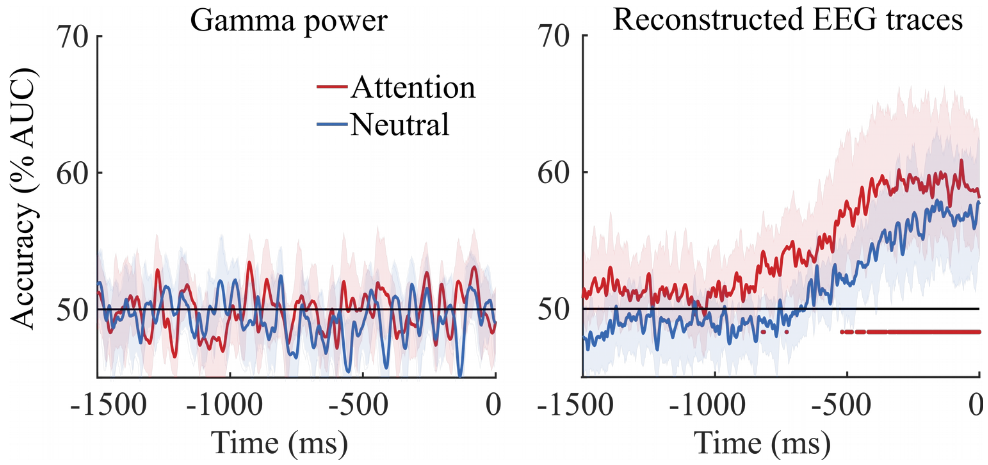

Figure 5

In addition to alpha oscillations also gamma band frequencies (> 30 Hz) have been associated with the inhibition and selection of sensory information (Bonnefond et Jensen 2013): distracters elicit a lower gamma power and a higher alpha power compared to target stimuli. Bonnefond et Jensen 2013 suggested that while alpha oscillations would reflect anticipative modulation of excitability,

gamma power would be linked to the interaction between this top-down modulation and the stimulus-driven activity. However, in the present study we were not able to decode the orientation of spatial attention from gamma power, suggesting that gamma oscillations did not contribute to classification accuracy of raw EEG traces.

Individual data: EEG classifiers

The graphs below depict the classifier accuracy (alpha, 10, 50 and 100 ms classifiers decoding left and right target trials) for each participant (i.e. 13 subjects) and condition (neutral and attention).

Alpha power classifier

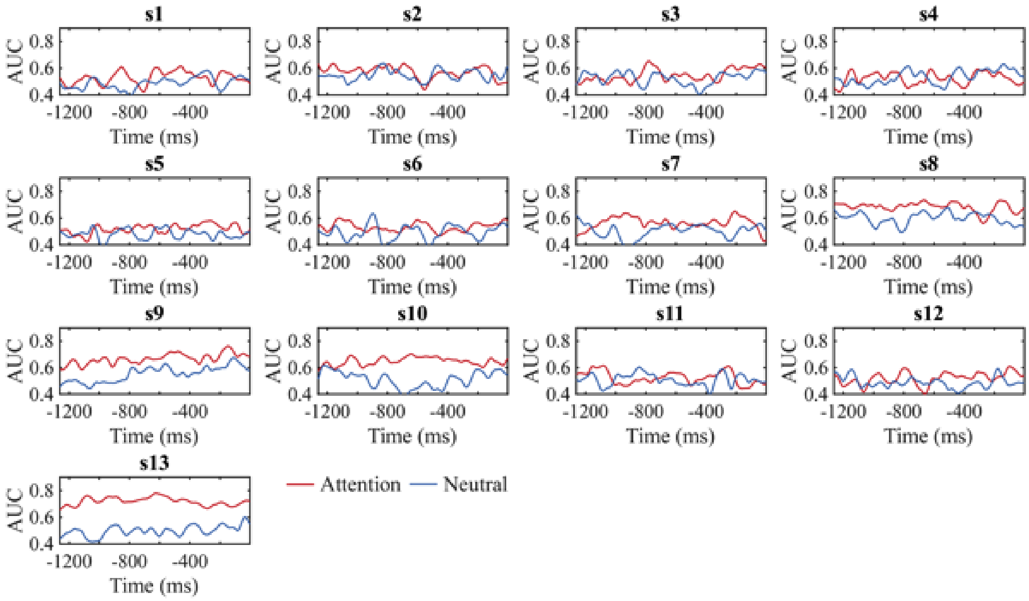

100 ms raw activity classifier

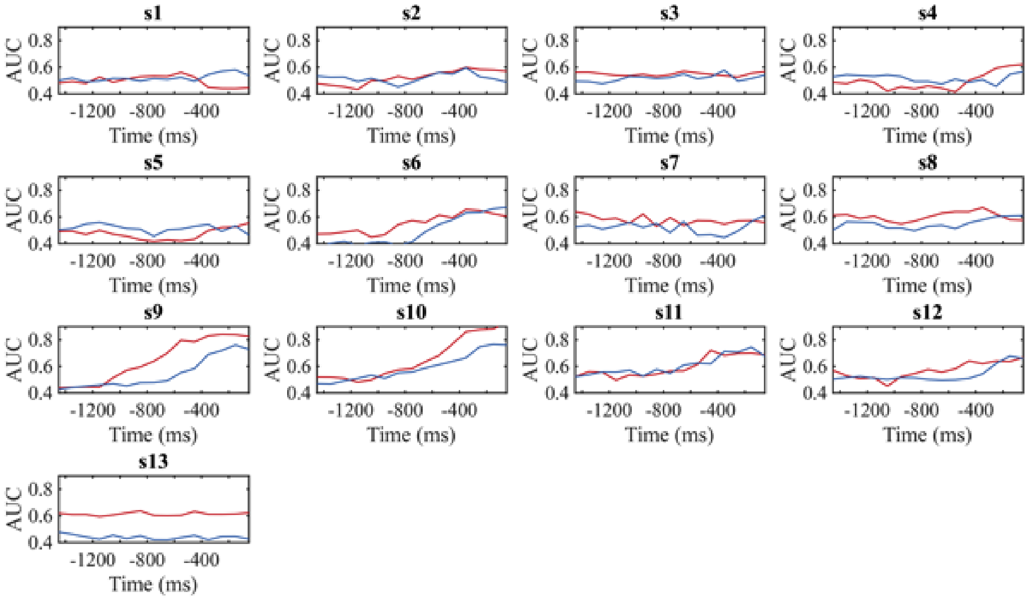

50 ms raw activity classifier

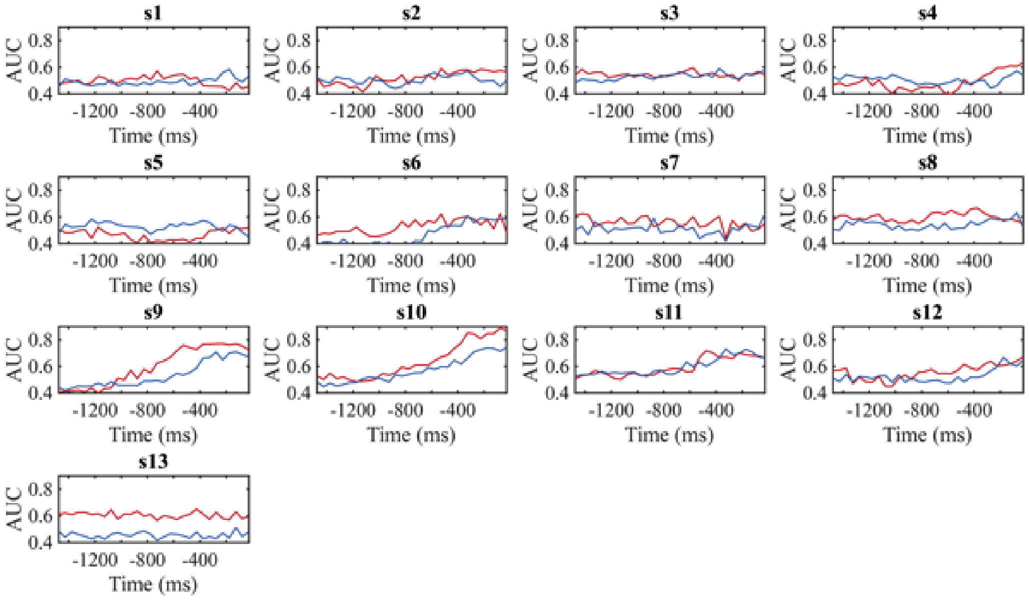

10 ms raw activity classifier

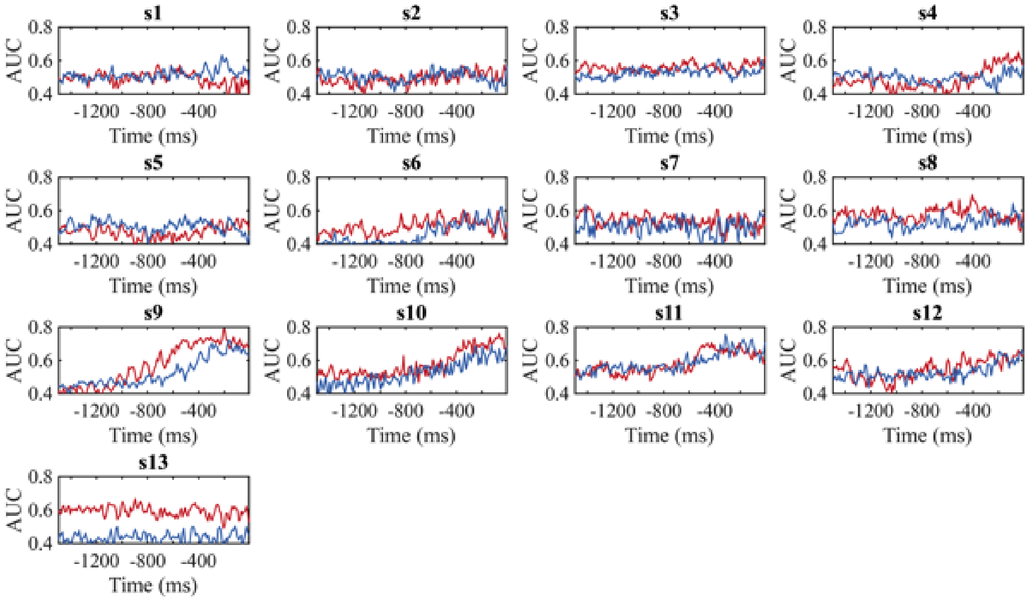

Supplement: Supplementary file 1 [file Data_Sheet_2.PDF]
